# Systematic Review and Meta-Analysis of the Prevalence and Risk Factors Associated With the Occurrence of Incisional Hernia in Patients Undergoing Midline Laparotomy

**DOI:** 10.3389/jaws.2026.15439

**Published:** 2026-03-06

**Authors:** Edgard Efren Lozada Hernandez, Luis Alberto Fernandez Vázquez-Mellado, Luis A. Martin-del-Campo, Héctor Ali Valenzuela Alpuche, Enrique Ricardo Jean Silver, H. Alejandro Rodríguez, Ricardo Reynoso González, Tatiana Andrea Prado Salcedo, Monserrat Martinez-Zamorano, Cesar Felipe Pleoneda Valencia

**Affiliations:** 1 Hospital Regional de Alta Especialidad del Bajio-IMSS Bienestar, Leon, Mexico; 2 Hospital Angeles Queretaro, Santiago de Querétaro, Mexico; 3 Hospital Angeles del Carmen, Guadalajara, Mexico; 4 Hospital Angeles Andares, Guadalajara, Mexico; 5 Asociacion Medica del Centro Medico ABC, Mexico City, Mexico; 6 Tecnologico de Monterrey Escuela de Medicina y Ciencias de la Salud, Monterrey, Mexico; 7 Hernia Care Center, Ciudad de Mexico, Mexico

**Keywords:** prevalence, incisional hernia, meta-analyses, midline laparotomy, risk factor

## Abstract

**Introduction:**

Incisional hernia (IH) is the main long-term complication after midline laparotomy and has significant clinical and economic effects. Although multiple risk factors for IH formation have been proposed, their ranking and clinical relevance have not been clearly established. This meta-analysis aimed to estimate the prevalence of IH and rank the associated risk factors, considering both their statistical significance and their clinical impact.

**Methods:**

This meta-analysis was conducted in accordance with the Preferred Reporting Items for Systematic Reviews and Meta-Analyses (PRISMA) guidelines and registered in PROSPERO (CRD420251107739). Observational (cohort and cross-sectional) studies evaluating patients undergoing midline laparotomy with follow-up for IH were included. Clinical trials and studies involving a laparoscopic approach were excluded. The global prevalence of IH was calculated, and random effects models were used to identify risk factors associated with the occurrence of IH, whose associations are reported as hazard ratios (HRs) and 95% confidence intervals.

**Results:**

Twenty studies (n = 790,800 patients) were included, among whom the overall prevalence of IH was 10.1% (95% CI: 7%–15%). Only 10 studies analyzed relevant risk factors. The factors with the greatest clinical impact were reoperation during hospitalization (HR = 4.09) and surgical site infection (HR = 2.96). Other significant factors included emergency surgery, colon surgery, stoma creation, diabetes, and liver disease. Factors such as sex, obesity, or hypertension were not significantly associated with IH formation.

**Conclusions:**

Perioperative factors are key determinants of the occurrence of IH. The identification of such factors would allow prioritization of preventive interventions, such as the application of prophylactic meshes, especially in high-risk patients. Standardized prospective studies are needed to validate these findings.

## Introduction

The main complication after midline laparotomy is incisional hernia (IH), with an prevalence of 12.8% at 23.8 months of follow-up [1, 2], although values of up to 69% have been reported in high-risk groups [3]. This prevalence is related to the study population evaluated, the type of incision made, and the methods of follow-up and with which the hernia is diagnosed [3–5].

IH not only results in morbidity associated with chronic pain, functional limitations and deterioration in quality of life but is also associated with a substantial economic burden related to the need for reinterventions [6]. In the United States, the cost of care for this complication is 4 billion dollars per year; a cost reduction of 32 million dollars has been estimated for every 1% decrease in the prevalence of IH [7]. Taken together, these data justify efforts to prevent the occurrence of this complication [2].

There is no single determining risk factor for the occurrence of IH, and the effects of each of the factors are cumulative; hence, not all patients have the same risk of developing an IH [2, 8]. Although preventative efforts have focused on optimizing the surgical technique used to close the incision or the use of prophylactic meshes [3], IH nevertheless occurs more frequently in patients with predisposing factors such as obesity [9], surgical site infection [10], malnutrition, the use of immunosuppressants and chronic diseases such as liver disease. More concerningly, several of these factors are unmodifiable, at least in emergency surgery settings [11].

Despite the multiple studies that have addressed factors associated with the development of IH and developed predictive scores, the results have been heterogeneous and sometimes contradictory. This inconsistency refers to the variation in reported clinical significance for the same factor. For instance, regarding male gender, Ganesh et al. [12] report finding no association, whereas Rios-Diaz et al. [13] report male gender as a statistically significant risk factor. Similarly, for Body Mass Index (BMI), Ganesh et al. [12] identify a significant risk for BMI >25 kg/m^2^, whereas Tecce et al. [14] report significance only for BMI >30 kg/m^2^, finding no association for BMI <30 kg/m^2^. Some predictive models have shown clinical utility, but their applicability is limited by the lack of external validation, the inclusion of postoperative variables that are unavailable at the time of surgical decision-making, and the lack of stratification by type of patient or procedure [15].

The evidence on IH risk arises from heterogeneous study populations that combine different surgical approaches. Given the fundamental anatomical and pathophysiological differences between a full-length midline fascial incision and minimal-access port sites (which involve distinct risk profiles, prevention strategies, and even clinical guidelines), a synthesis focused specifically on open midline laparotomy is warranted. This approach ensures methodological homogeneity and provides clinically actionable evidence for the high-risk patient population that requires this specific surgical access, often in settings of emergency, complexity, or contraindication to minimally invasive techniques.

The objective of this study was to quantitatively evaluate the relative impact of relevant risk factors on the prevalence of IH after midline laparotomy through a meta-analysis of cohort studies to identify the specific magnitude of the effect of each factor. This analysis will establish a hierarchy of risk factors, providing solid evidence to guide clinical decision-making while also identifying the actual prevalence of IH associated with midline laparotomy.

The rationale for establishing this hierarchy is to move beyond a mere list of associations and create a clear, actionable framework for clinical practice. By stratifying risk, the findings can directly guide the intensity of preventive strategies: from mandating advanced measures like prophylactic mesh for high-risk patients to optimizing standard care for others. This approach personalizes management, prevents intervention fatigue, and focuses resources where they offer the greatest benefit.

## Methods

A meta-analysis was conducted and reported in accordance with the Preferred Reporting Items for Systematic reviews and Meta-Analyses (PRISMA) guidelines [16]. The study was registered with the hospital research and research ethics committees of the hospital, with registration numbers CEI/HRAEB/002/2025 and CEI-001-2025 and in the International Registry for Prospective Systematic Evaluations in PROSPERO (registration number: CRD420251107739) [17].

### Study Objectives

This meta-analysis had two primary objectives, formulated as specific research questions:

1. Research Question 1 (Risk Factors): What is the hierarchy of risk factors based on the magnitude of their effect, associated with the development of IH after midline laparotomy?
2. Research Question 2 (prevalence): What is the pooled cumulative prevalence of IH in adult patients after midline laparotomy?

### Eligibility Criteria (PEO Framework)

The eligibility criteria were structured according to the PEO framework (Population, Exposure, Outcome), which is the recommended format for systematic reviews addressing questions of risk factors and prevalence.

Population (P): Adult patients (>18 years) undergo midline laparotomy. Exposure (E): For the synthesis of risk factors, the “exposure” was defined as any pre-, intra-, or postoperative patient characteristic or surgical variable (e.g., demographics, comorbidities, technical factors). For the synthesis of prevalence, the “exposure” was the index surgical event (midline laparotomy). Outcome (O): The primary outcome was the diagnosis of an IH during the postoperative follow-up period, confirmed clinically or radiologically [18].

### Inclusion and Exclusion Criteria

#### Inclusion Criteria

*Study population*: Patients who underwent midline laparotomy, regardless of surgical indication (urgent or elective), with postoperative follow-up for the detection of IH. *Type of study*: Cohort observational studies (prospective or retrospective) and cross-sectional studies were included. *Study content*: Studies that clearly reported the prevalence of IH as well as the associated risk factors for the condition (demographic, clinical, nutritional or technical). Language: Studies published in English with available full text.

#### Exclusion Criteria

Studies focused on other types of surgical approach (exclusively laparoscopic or robotic) or another type of incision (transverse, paramedian). Studies including patients with a preexisting IH before the first surgery. Studies that did not report relevant data on the prevalence of IH or its risk factors. Nonprimary publications such as narrative reviews, systematic reviews, previous meta-analyses, conference abstracts, letters to the editor, clinical guidelines, theses or institutional reports.

### Data Sources and Search Terms

A systematic search was carried out in the following databases for studies published in English between 1 January 2000, and 30 June 2025: PubMed, The Cochrane Library, SCOPUS, ScienceDirect, ProQuest and Google Scholar.

A combination of controlled terms (MeSH) and free terms was used to maximize the sensitivity of the search strategy. In addition, the bibliographic references of the included studies were manually reviewed to identify relevant articles that may have been omitted in the automated search. The search terms included: “Incisional Hernia,” “Risk Factor,” “Influencing Factor,” “Associated Factor,” “Laparotomy,” and “Abdominal Surgery.” The full search strategy is described in Annex 1, Supplementary Table A.

### Selection of Studies and Quality Assessment

Two investigators independently (L.A.M.C. and H.V.A.) conducted the study selection and data extraction process. The titles and abstracts were subsequently reviewed for preliminary study selection. Finally, the selected texts were read completely to determine their eligibility according to the previously defined inclusion and exclusion criteria. Disagreements were resolved by discussion among the investigators, and if they persisted, a third evaluator (L.A.F.V.M) was consulted to make the final decision. Information extracted from each study included the name of the first author, year of publication, country of origin, type of study, characteristics of the participants, total sample size, number of patients with IH and reported risk factors.

The risk of bias in the studies included was assessed independently by two investigators (E.R.J.S. and H.A.R.). For non-randomized cohort studies, the investigators used the Risk Of Bias In Non-randomized Studies – of Interventions (ROBINS-I) tool. This tool evaluates bias across seven domains: bias due to confounding, participant selection, classification of interventions, deviations from intended interventions, missing data, measurement of outcomes, and selection of the reported result. Each study received an overall judgment of Low, Moderate, Serious, or Critical risk of bias (Supplementary Figure SA,B) [19, 20].

### Data Extraction and Management

Data extraction was performed using a form developed in Microsoft Excel, applied independently by two investigators. The extracted data was organized into the following categories:

Study and Population Characteristics: Including author, year, design, sample size, number of incisional hernia (IH) cases, and prevalence (columns 1-7, Table 1).

**TABLE 1:** Characteristics of the studies included (n = 20).

Author, Year	Country	Study type	IH/Simple size	Prevalence (95% CI)	Follow-up (months)	Risk of Bias (ROBINS-I)
Ganesh et al. [12]	India	Cross-sectional	18/100	18 (11–26)	8	7/9 High Quality*
Fink et al. [4]	Germany	Prospective cohort	173/775	22.3(19.4–25.3)	36	7.5/9 High Quality
Rios-Diaz et al. [13]	USA	Retrospective cohort	3127/35666	8.7(8.4–9.06)	67	9/9 High Quality
Veljkovic et al. [9]	USA	Prospective cohort	81/522	15.5(12.4–18.6)	7	8/9 High Quality
Walming et al. [10]	Sweden	Retrospective cohort	166/1621	10.2(8.7–11.7)	12	8.5/9 High Quality
Basta et al. [21]	USA	Retrospective cohort	42/497	8.4 (6–10.9)	28.3	8.5/9 High Quality
Basta et al. [22]	USA	Retrospective cohort	1398/19902	7 (6.66–7.38)	57.9	8/9 High Quality
Ortega-Deballon et al. [23]	France	Retrospective cohort	22944/672429	3.4(3.3–3.5)	60	9/9 High Quality
Cherla et al. [24]	USA	Retrospective cohort	114/247	46.2(39.9–52.3)	21.4	9/9 High Quality
Moas et al. [25]	USA	Retrospective cohort	109/570	19.1(15.9–22.3)	24	8/9 High Quality
Tecce et al. [14]	USA	Retrospective cohort	59/852	6.9(5.2–8.6)	35.5	9/9 High Quality
Franchi et al. [26]	Italy	Retrospective cohort	77/455	16.9(9.5–24.2)	120	8/9 High Quality
Lozada et al. [17]	Mexico	Retrospective cohort	161/789	20.4(17.5–23.2)	24	7/9 High Quality
Fisher et al. [27]	USA	Retrospective cohort	436/12373	3.5 (3.1–3.8)	32.2	9/9 High Quality
Goodenough et al. [28]	USA	Prospective cohort	93/625	14.8(12–17.6)	41	8/9 High Quality
Weissler et al. [29]	USA	Retrospective cohort	2563/30741	8.3(8–8.6)	24	9/9 High Quality
Höer et al. [30]	Germany	Retrospective cohort	128/2983	4.2(3.5–5)	120	8/9 High Quality
Tansawet et al. [11]	Thailand	Retrospective cohort	101/5431	1.8(1.5–2.2)	23.4	9/9 High Quality
Adell-Carceller et al. [31]	Spain	Retrospective cohort	43/295	10.2(8.7–11.7)	37.5	8/9 High Quality
Itatsu et al. [32]	Japan	Retrospective cohort	318/3927	8.1(7.2–8.9)	24	8/9 High Quality

Evaluated using the scale proposed by the Agency for Healthcare Research and Quality (AHRQ), the remaining studies were assessed with the Newcastle-Ottawa Scale (NOS).

Definitions: The operational definition of IH and the follow-up method were synthesized narratively.

Data for Meta-Analysis: For prevalence, data on cases and sample size from Table 1 were used. For risk factors, raw data were extracted (number of patients with/without IH who had each factor), constructing 2 × 2 tables, as the Hazard Ratio (HR) was not uniformly reported. For continuous variables, the mean and standard deviation were extracted as reported by the primary studies. For age, the cutoff of ≥65 years was applied in analyses where studies dichotomized this variable. For BMI, the WHO definition for obesity (≥30 kg/m^2^) was used as the standard cutoff in our synthesis. We did not test the distribution of these variables, as individual patient data was not available.

Discrepancies were resolved by consensus or, when necessary, by a senior third investigator. The synthesis for each risk factor was performed using only the subset of studies that reported the necessary raw data for that specific factor; therefore, the number of studies (k) varied across factors. Finally, the data was organized in the R environment (version 4.3.0) for analysis.

### Main Outcomes

Primary Outcome: The strength of association between potential risk factors and the occurrence of incisional hernia (IH), measured as the Hazard Ratio (HR) with its 95% confidence interval. The definition of IH (e.g., clinical diagnosis, imaging confirmation, or surgical repair) was based on the criteria reported in each primary study and was recorded during the data extraction process.

Secondary Outcome: The cumulative prevalence of IH after midline laparotomy, calculated as the proportion of patients who developed a hernia relative to the total surgical cohort.

### Statistical Analysis

A meta-analysis was conducted to estimate the cumulative prevalence of IH after midline laparotomy and to identify the risk factors associated with its formation. Hazard Ratios (HRs) with their 95% confidence intervals (CIs) were calculated for all risk factors, irrespective of whether they were continuous (e.g., age) or categorical (e.g., presence of diabetes) variables. The effect sizes were synthesized using a random-effects model (Der Simonian and Laird method) to incorporate the anticipated variability between studies. Heterogeneity among the included studies was assessed using the Cochrane Q statistic and the Higgins and Thompson I^2^ index. Substantial heterogeneity was considered present if I^2^ ≥ 50% alongside a p-value ≤0.05 for the Q statistic. Sensitivity analyses were conducted to assess the robustness of the pooled estimates. First, we performed a leave-one-out analysis by sequentially excluding each study. Second, we compared the results from our primary random-effects model with those obtained from a fixed-effects model. Subgroup analyses were carried out to explore possible sources of heterogeneity, such as differences in the population or methodological characteristics of the included studies. To evaluate the existence of publication bias, a funnel plot was generated, and Egger’s test was performed. P < 0.05 was considered to indicate statistical significance. All the statistical analyses were performed in the R Studio environment (version 1.4.1106) using the R language (version 4.3.0) with the meta, metafor, dmetar and metasens packages.

## Results

### Study Selection

**FIGURE 1:**
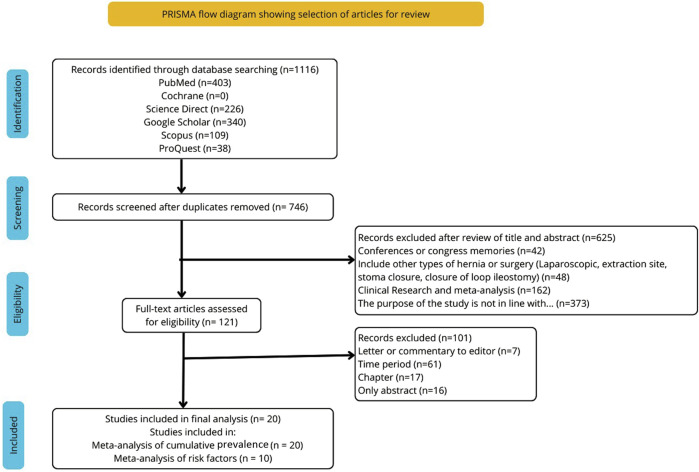
Preferred Reporting Items for Systematic Reviews and Meta-Analyses (PRISMA) flowchart of literature search and selection.

Following a search of the abovementioned databases, a total of 1,116 articles related to the topic were found. A total of 370 duplicate articles were removed, followed by 625 following the initial review. Among the 101 articles assessed for eligibility, 20 were included in the quantitative synthesis for the cumulative prevalence of IH. Of these, 10 studies provided data in an extractable format suitable for the meta-analysis of risk factors [4, 9–14, 21–33]. The study selection flowchart is shown in Figure 1.

### Study Characteristics

**FIGURE 2:**
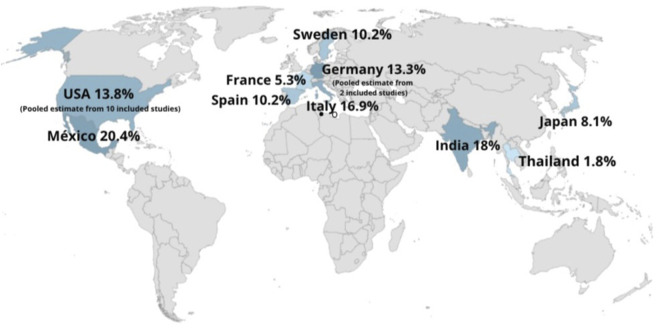
Global prevalence of IH.

The countries of origin of the included studies were as follows: the United States [10], Germany [2], India, France, Italy, Mexico, Sweden, Spain, Japan and Thailand (one study each) (Figure 2). The methodologies of the studies were distributed as follows: 16 retrospective cohort studies, 3 prospective cohort studies and 1 cross-sectional study. The risk of bias assessment for the cohort studies, conducted using the ROBINS-I tool, indicated that most studies (n = 16) were judged to be at moderate risk of bias. Three studies [3] were assessed as having a low risk of bias, and one study [1] was judged to be at high risk of bias. A detailed summary is provided in Supplementary Table C.

### Prevalence Meta-Analysis

**FIGURE 3:**
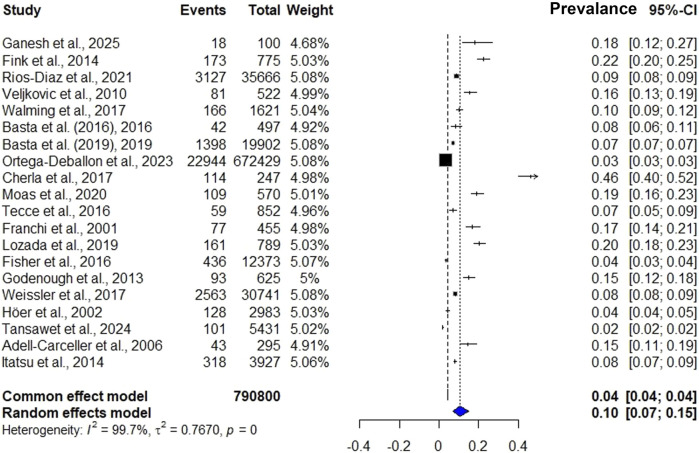
Meta-analysis of the prevalence of IH.

In total, 790,800 patients who underwent laparotomy were included in the analysis, of whom 32,151 developed IH. The mean prevalence was 10.1% (95% CI 7%–15%), with a range of 1.8%–46.2%, and the mean follow-up was 39.6 months (Figure 3). The general characteristics of the included studies are presented in Table 1.

#### Sensitivity Analysis and Publication Bias

A sensitivity analysis was performed by sequentially excluding studies (the “*leave-one-out*” method). This analysis revealed that the pooled prevalence remained stable, with values that ranged between −2.4 and −2.1 on the logit scale (equivalent to ∼8.2%–11.3% on the natural scale); this value was not significantly different from the original estimate (−2.2 logit; 95% CI: [−2.3, −2.1]). The exclusion of any one study substantially altered the results (i.e., all confidence intervals overlapped with the original value), suggesting that the conclusions of the meta-analysis are robust and do not depend on a particular study (Annex 1, Supplementary Figure SA). The analysis revealed the presence of publication bias (p = 0.002) and high heterogeneity, suggesting that the results should be interpreted with caution. Although the direction of the effect was consistent, the variability between studies limits the generalizability of the estimates (Annex 1, Supplementary Figure B).

#### Sensitivity Analysis by Region and Type of Study

A subanalysis was performed by geographic region, revealing the highest prevalence in Europe (13.4%), followed by Asia (10.4%) and North America (9.8%). In terms of the type of study, a higher prevalence was found among prospective studies (15.2%) than among retrospective studies (9.5%). All these analyses involved studies with >50% heterogeneity and thus were interpreted with a random effects model (Table 2).

**TABLE 2:** Pooled prevalence estimates with subgroup analyses.

1. By geographic region						
Region	Studies (n)	I^2^ (%)	p (Heterogeneity)	Model	Prevalence (%)	95% CI
Asia	3	76.6	<0.001	Random effects	10.4	7.0–13.9
North America	11	94.2	<0.001	Random effects	9.8	6.3–13.3
Europe	5	91.2	<0.001	Random effects	13.4	7.8–19.1

2. By study design						
Study type	Studies (n)	I^2^ (%)	P (heterogeneity)	Model	Prevalence (%)	95% CI
Prospective	3	85.4	<0.001	Random effects	15.2	8.7–21.7
Retrospective	16	93.1	<0.001	Random effects	9.5	7.2–11.8

The North American subgroup comprises studies from the United States and Mexico. The single cross-sectional study was omitted from subgroup comparisons due to insufficient sample size.

### Meta-Analysis of Risk Factors

The meta-analysis of prevalence included all 20 available studies. However, for the analysis of risk factors, 10 studies were excluded: three because they did not compare groups with and without IH [4, 26, 31] and seven because, although they reported general prevalence by surgical approach (and thus were included in the meta-analysis of prevalence), they did not provide separate data for the analysis of risk factors by the type of surgical approach [11, 21, 22, 24, 27, 28, 32]. Studies with mixed approaches were excluded because the objective of this study was to evaluate patients undergoing midline laparotomy.

Among the ten included studies, 76 variables related to the occurrence of IH were identified. Of these, 35 were excluded because they did not meet the inclusion criteria (for example, cost and type of hospital), and 21 were excluded because they were reported in only one or two studies, which made it impossible to meta-analyze them (e.g., cough and use of steroids). Finally, 20 variables were considered for analysis (Supplementary Table B). The variables included were grouped into three categories: demographic factors, comorbidities, and perioperative factors.

Demographic factors: Sex: No statistically significant differences were found between men and women. Age: The association between age and IH risk was analyzed separately based on how the data were reported in the primary studies. In studies reporting age as a continuous variable (mean and standard deviation), a significant positive association was found (HR = 1.11; 95% CI: 1.06–1.15). Similarly, in studies that dichotomized age using a cutoff of ≥65 years, older age was significantly associated with a higher risk of IH (HR = 1.28; 95% CI: 1.26–1.30). Thus, regardless of the analytical approach, increased age was consistently identified as a significant risk factor for IH.

Body mass index (BMI): Studies reported BMI continuously or with different cutoff points (≥25 or ≥30 kg/m^2^). For this analysis, a cutoff point of ≥30 kg/m^2^ was chosen to define obesity, but the association with the incidence of IH was not significant.

Comorbidities: In the analysis of comorbidities associated with the presence of IH, diabetes (HR = 1.63; 95% CI: 1.11–2.41), smoking (HR = 1.40; 95% CI: 1.12–1.74), chronic lung disease (HR = 1.30; 95% CI: 1.20–1.36), liver disease (HR = 1.76; 95% CI: 1.50–2.06) and kidney failure (HR = 1.20; 95% CI: 1.14–1.27) were significantly associated with an increased risk of developing IH.

In contrast, the presence of hypertension (HR = 1.18; 95% CI: 0.95–1.48), chronic heart failure (HR = 1.06; 95% CI: 0.72–1.57), or anemia (HR = 1.00; 95% CI: 0.84–1.20) or a diagnosis of cancer (HR = 1.38; 95% CI: 0.99–1.93) was not significantly associated with the occurrence of IH.

**FIGURE 4:**
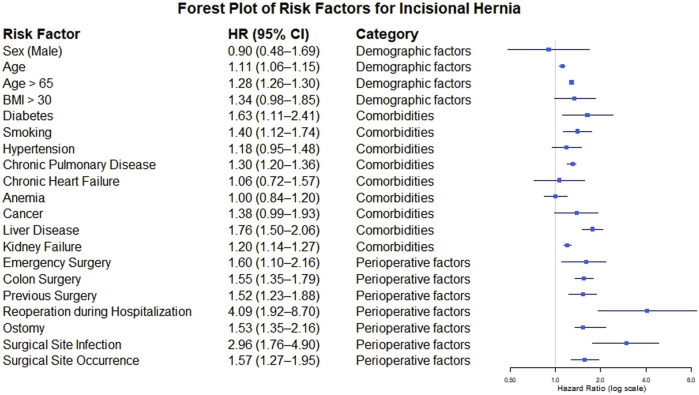
Forest plot risk factors of IH.

Perioperative factors included emergency surgery (HR = 1.60; 95% CI: 1.10–2.16)), colon surgery (HR = 1.55; 95% CI: 1.35–1.79), a history of previous surgery (HR = 1.52; 95% CI: 1.23–1.88), reoperation during hospitalization (HR = 4.09; 95% CI: 1.92–8.70), creation of a stoma during surgery (HR = 1.53; 95% CI: 1.35–2.16), the presence of surgical site infection (HR = 2.96; 95% CI: 1.78–4.90) and surgical site ocurrence (including infection) (HR = 1.57; 95% CI: 1.27–1.95). All these factors were significantly associated with the development of IH; the ones with the greatest impact were reoperation during hospitalization and the presence of surgical site infection (Table 3; Figure 4).

**TABLE 3:** Results of meta-analysis of risk factors for incisional hernia.

Risk factor	Publications	Effect model	Meta-analysis results	Heterogeneity
			HR (CI95%)	I^2^/p
Demographic factors				
Sex male	6	Random effects model	0.9 (0.48–1.69)	98%/<0.0001
Age	5	Random effects model	1.11 (1.06–1.15)	96%/<0.0001
Age >65	7	Fixed effects model	1.28 (1.26–1.30)	0%/0.69
BMI >30	8	Random effects model	1.34 (0.98–1.85)	​
Comorbidities				
Diabetes	7	Random effects model	1.63 (1.11–2.41)	96%/<0.0001
Smoking	4	Random effects model	1.4 (1.12–1.74)	70.5%/0.01
Hypertension	3	Random effects model	1.18 (0.95–1.48)	85.4%/0.001
Chronic Pulmonary disease	6	Fixed effects model	1.3 (1.2–1.36)	0%/0.52
Chronic heart failure	5	Random effects model	1.06 (0.72–1.57)	96.9%/<0.0001
Anemia	5	Random effects model	1 (0.84–1.20)	72.5%/0.0058
Cancer	4	Random effects model	1.38 (0.99–1.93)	88.5%/<0.0001
Liver disease	5	Random effects model	1.76 (1.5–2.06)	59.4%/0.043
Kidney failure	6	Fixed effects model	1.2 (1.14–1.27)	1.1%/0.4
Perioperative factors				
Emergency surgery	3	Fixed effects model	1.6 (1.1–2.16)	31.4%/0.23
Colon surgery	4	Random effects model	1.55 (1.35–1.79)	90.1%/<0.0001
Previous surgery	4	Fixed effects model	1.52 (1.23–1.88)	36.5%/0.19
Reoperation during hospitalization	4	Random effects model	4.09 (1.92–8.7)	98.7%/<0.0001
Ostomy	4	Fixed effects model	1.53 (1.35–2.16)	29.4%/0.23
Surgical site infection	7	Random effects model	2.96 (1.76–4.9)	90.3%/<0.0001
Surgical site ocurrence	3	Random effects model	1.57 (1.27–1.95)	69.6%/0.037

BMI: Body mass index. I^2^: percentage of heterogeneity. p: p value. HR: Hazard ratio. CI: confidence interval.

## Discussion

The European Hernia Society (EHS) guidelines for abdominal wall closure, published in 2015 and updated in 2022, report an prevalence of IH of 12.8% [2, 3] on the basis of the results of a meta-analysis by Bosanquet et al. (2015) [1]. However, that study presents several methodological biases that must be considered. First, 48% of the studies included were clinical trials, a design that is not ideal for estimating prevalence, since it is limited by the population defined by the inclusion criteria (for example, patients with obesity or aortic aneurysm, which does not represent the general population) [34, 35].

Second, 44% of the clinical trials included involved use of a laparoscopic approach. Pooling data from both open and laparoscopic procedures introduces substantial clinical heterogeneity and potential confounding, since the prevalence of IH differs substantially between these approaches, a fact recognized by the EHS guidelines, which state that laparoscopy reduces IH risk. Therefore, the aggregated prevalence reported by Bosanquet et al. may not accurately reflect the risk specific to open surgery. In contrast, by design, our meta-analysis focused exclusively on open midline laparotomy, and by excluding studies involving laparoscopic approaches, our pooled prevalence estimate is specific to the open midline laparotomy population. Consequently, our pooled prevalence estimate (10.1%) provides a more precise and directly applicable measure of risk for the specific patient population undergoing open abdominal surgery.

Finally, 38% of the included studies were conducted in the 1980s and 1990s, when both the imaging methods and the surgical techniques used substantially differed from those currently in use, which could have led to overestimations of the reported prevalence.

In this study, we reported an prevalence of IH of 10.1% at 39.6 months of follow-up. Only data from cohort and cross-sectional studies were analyzed; clinical trials and studies involving laparoscopic approaches were excluded. Consequently, our estimate specifically reflects the risk in patients undergoing open surgery. However, two important factors should be noted in the evaluation of this result: 1. Among the included studies, some involved diagnoses obtained from the patients’ files, while in others, patients who underwent IH repair were identified during the study period. Consequently, one potential bias is related to the fact that 16%–44% of patients with IH are usually asymptomatic [24, 36]; additionally, it has been reported that among patients with hernia, only 6.6%–20% end up undergoing surgical repair [26, 32, 37]. 2. Analyses of retrospective and prospective studies yielded incidence values of 9.5% vs. 15.2%, respectively; in the prospective studies, if physical examinations or imaging studies were performed to corroborate the presence of IH, performing imaging studies increased the number of patients identified with IH by up to 40% [24, 38].

One of the main objectives of this study was to rank the risk factors associated with the development of IH on the basis of not only their statistical significance but also their real clinical impact. In this sense, although the associations for multiple variables reached statistical significance (p < 0.05), the magnitude of the effect, measured with the HR, allowed us to determine the differences in the clinical impact of these factors. The factors with the highest risk (HR >2.0) included reoperation during hospitalization (HR = 4.09) and surgical site infection (HR = 2.96). Factors that imparted moderate–high risk (HR 1.5–2.0) included liver disease (HR = 1.76), diabetes (HR = 1.63), emergency surgery (HR = 1.60), surgical site complications (HR = 1.57), colon surgery (HR = 1.55), stoma creation (HR = 1.53) and a history of previous surgery (HR = 1.52). Factors that imparted moderate–low risk (HR 1.2–1.5) included smoking (HR = 1.40), chronic lung disease (HR = 1.30), kidney failure (HR = 1.20) and age ≥65 years (HR = 1.28). Finally, there was no significant association between sex, BMI ≥30 kg/m^2^, hypertension (HR = 1.18), chronic heart failure (HR = 1.06), anemia (HR = 1.00) or cancer (HR = 1.38) and IH development.

The literature mentions that an HR >1.5 is usually considered the threshold of clinical relevance, since it represents a substantial increase in risk that justifies intervention. In contrast, an HR between 1.1 and 1.2, although potentially statistically significant, implies only a 10%–20% increase in risk, which rarely merits changes in clinical practice. In this study, factors such as age ≥65 years (HR = 1.28) and kidney failure (HR = 1.20) fell into this category and thus had limited clinical significance. In contrast, a hazard ratio (HR) >1.5 (and especially >2.0), such as that for reoperation and surgical infection, identified the variable as a priority target around which preventive strategies could be developed. This distinction is crucial to avoid over intervention based on marginal associations and focus resources on factors that truly impact the risk of IH [39, 40].

The impact of surgical site infection on the occurrence of IH is significant. Infections can compromise the integrity of the surgical wound and weaken the tissue and fascia at the incision site, which can lead to poor healing. In addition, surgical site infections can prolong hospitalization time, increase the need for additional surgical interventions to treat hernia and infection, and increase healthcare costs [41, 42]. This association is highly relevant, yet it has been underutilized in risk prediction. Few existing scales for predicting IH incorporate SSI as a predictor, and those that do often treat it simply as a dichotomous variable. Given that SSI emerged as the strongest risk factor in our analysis and considering that validated scales already exist to predict SSI, integrating these SSI prediction tools into existing or novel IH risk scores could significantly improve their predictive performance [43].

This meta-analysis presents several relevant strengths. The inclusion of studies from different regions and a cumulative sample of almost 800,000 patients (including more than 32,000 cases of incisional hernia), allows the estimation of the prevalence of IH in patients undergoing midline laparotomy with high precision. Only observational studies (multiple cohort and one cross-sectional study) were analyzed, none of which mixed experimental designs or different surgical approaches, which improves the clinical applicability of the results. In addition, the sensitivity analysis demonstrated the robustness of the estimates, and the protocol was registered in PROSPERO according to the PRISMA guidelines.

This review has limitations inherent to its design and the included studies. First, the assessment using the ROBINS-I tool indicated that potential selection bias (D2) was present across studies, as illustrated in Supplementary Appendix 3, which may affect the generalizability of our pooled estimates. Second, our evaluation of publication bias using Egger’s test yielded a statistically significant result (p = 0.002), and the accompanying funnel plot (Supplementary Figure SB) showed asymmetry, suggesting a potential underrepresentation of smaller studies with null or negative findings. Although we employed a comprehensive search strategy, this asymmetry indicates that the overall effect size should be interpreted with caution, as the meta-analytic estimate might lead to an overestimation of the true effect.

This study also has several limitations. One of these is the high heterogeneity among studies, which can be attributed to differences in definitions, diagnostic methods, and designs. Another important limitation is the relatively short mean follow-up of 39.6 months across the included studies, as IH can develop later than this period. Only ten of the twenty included studies contributed data for the risk factor analysis, which restricted the number of evaluable variables. Among the 76 initially identified factors, only 20 could be analyzed in an aggregate manner. Most of the studies were retrospective in nature, which is associated with a high risk of bias, and publication bias was evident. Some clinically relevant factors could not be included because of a lack of consistent data between studies. A major and modifiable limitation is the absence of data on fascial closure technique (e.g., suture-to-wound length ratio, stitch size), a critical peri-operative variable known to profoundly influence IH risk, which was not reported in the observational studies we synthesized.

These limitations reinforce the need for multinational prospective studies, with standardized protocols that validate the identified factors and allow exploration of other factors that have not yet been analyzed in sufficient depth.

## Conclusions

This study revealed that IH after midline laparotomy is a frequent complication, with an estimated prevalence of 10.1%. Perioperative factors, especially in-hospital reoperation and surgical site infection, showed the greatest clinical relevance, surpassing even multiple comorbidities in terms of the imposed risk. The ranking of these factors according to their clinical impact could allow a more precise development of prevention strategies. The findings of this study underscore the need for standardized prospective studies that validate and complement this evidence to improve decision-making in abdominal surgery.
