# Supplementary material for: Systematic Review and Meta-Analysis of the Prevalence and Risk Factors Associated With the Occurrence of Incisional Hernia in Patients Undergoing Midline Laparotomy
Source: J Abdom Wall Surg. 2026 Mar 6;5:15439. doi: 10.3389/jaws.2026.15439 (PMC13002507; doi:10.3389/jaws.2026.15439)

**Appendix 1**

**Table A1.** PubMed Search Strategy

| Number |  | Search Formula |
| --- | --- | --- |
| #1 |  | Laparotomy [Mesh] |
| #2 |  | Laparotomy [Title/Abstract] OR Midline Incision [Title/Abstract] OR Midline Laparotomy [Title/Abstract] OR Abdominal Incision [Title/Abstract] |
| #3 |  | #1 OR #2 |
| #4 |  | Incisional Hernia [Mesh] |
| #5 |  | Incisional Hernia*[Title/Abstract] OR Hernia*, Incisional [Title/Abstract] OR Postoperative Hernia*[Title/Abstract] OR Surgical Hernia*[Title/Abstract] |
| #6 |  | #4 OR #5 |
| #7 |  | Risk Factor*[Title/Abstract] OR Influencing Factor*[Title/Abstract] OR Associated Factor*[Title/Abstract] OR Predictive Factor*[Title/Abstract] |
| #8 |  | #3 AND #6 AND #7 |

**Table A2**. Search Strategies by Database

| Database |  |  |  |
| --- | --- | --- | --- |
| Web of Science | TS=(laparotomy OR "midline incision" OR "midline laparotomy" OR "abdominal incision") AND TS=("incisional hernia" OR "postoperative hernia" OR "surgical hernia") AND TS=("risk factor" OR "influencing factor" OR "associated factor" OR "predictive factor") | |  |
| SCOPUS | TITLE-ABS-KEY(laparotomy OR "midline incision" OR "midline laparotomy" OR "abdominal incision") AND TITLE-ABS-KEY("incisional hernia" OR "postoperative hernia" OR "surgical hernia") AND TITLE-ABS-KEY("risk factor" OR "influencing factor" OR "associated factor" OR "predictive factor") | |  |
| The Cochrane Library | ("laparotomy" OR "midline incision" OR "midline laparotomy" OR "abdominal incision") AND ("incisional hernia" OR "postoperative hernia" OR "surgical hernia") AND ("risk factor" OR "influencing factor" OR "associated factor" OR "predictive factor") | |  |
| ScienceDirect | ("laparotomy" OR "midline incision" OR "midline laparotomy" OR "abdominal incision") AND ("incisional hernia" OR "postoperative hernia" OR "surgical hernia") AND ("risk factor" OR "influencing factor" OR "associated factor" OR "predictive factor" | |  |
| ProQuest | TI,AB,SU (laparotomy OR "midline incision" OR "midline laparotomy" OR "abdominal incision") AND TI,AB,SU ("incisional hernia" OR "postoperative hernia" OR "surgical hernia") AND TI,AB,SU ("risk factor" OR "influencing factor" OR "associated factor" OR "predictive factor") | |  |
| MEDLINE (via Ovid and EBSCOhost) | (laparotomy.mp. OR "midline incision".mp. OR "midline laparotomy".mp. OR "abdominal incision".mp.) AND ("incisional hernia".mp. OR "postoperative hernia".mp. OR "surgical hernia".mp.) AND ("risk factor".mp. OR "influencing factor".mp. OR "associated factor".mp. OR "predictive factor".mp.) | |  |
| Google Schoolar | "laparotomy" OR "midline incision" OR "midline laparotomy" OR "abdominal incision" AND "incisional hernia" OR "postoperative hernia" AND "risk factor" OR "associated factor" | |  |

**Table B.** Variables not included

**Excluded because did not meet inclusion criteria**

| **Study** | **Variables Excluded** |
| --- | --- |
| Rios-Diaz et al., 2021 | Race, Injury severity score, Mechanism of injury, Insurance, Income, State |
| Walming et al., 2017 | Suture technique |
| Ortega-Deballon et al., 2023 | Index Hospital Duration, Index hospital status, Cost, Time between index surgery and first hernia repair, Volume activity of the hospital |
| Moas et al., 2020 | Mechanism of injury, Delayed closure of abdominal wound, Other suture of abdominal closure |
| Tecce et al., 2016 | Race, Cost, Readmissions |
| Franchi et al., 2001 | Junior or senior surgeon, Duration of surgery, Fascial closure technique |
| Fisher et al., 2016 | Index case LOS days, Readmissions, Cost, Race |

**Excluded because analyzed in only one or two studies (not suitable for meta-analysis)**

| **Study** | **Variables Excluded** |
| --- | --- |
| Ganesh et al., 2025 | Duration >2hrs surgery, Location, Defect size |
| Rios-Diaz et al., 2021 | Coagulopathy, Weight loss, Charlson Index |
| Veljkovic et al., 2010 | Transverse Diameter, Mean fascial incision length, Mean length of fascial suture, Mean ratio: fascial suture length to sagittal abdominal diameter, Mean ratio: fascial suture length to fascial incision length, Mean postoperative nausea score, Mean postoperative emesis score, Mean postoperative cough score, Mean postoperative pain score, Mean duration postoperative analgesic, Mean duration postoperative antibiotic, Mean time to suture removal or complete epithelialization |
| Walming et al., 2017 | Hypoalbuminemia, Peroral cortison |
| Tecce et al., 2016 | Hyperlipidemia, Malnutrition, History of wound complication, Pelvic prolapse, Fistula enterocutaneous, Operative small bowel obstruction |
| Franchi et al., 2001 | Mode of access to abdomen, Antibiotic prophylaxis |
| Lozada et al., 2019 | Cough, Wound greater than 18 cm |

**Appendix 2. Figures sensitivity analysis and funnel plot**

**Figure A.**  Sensitivity analysis of IH rates


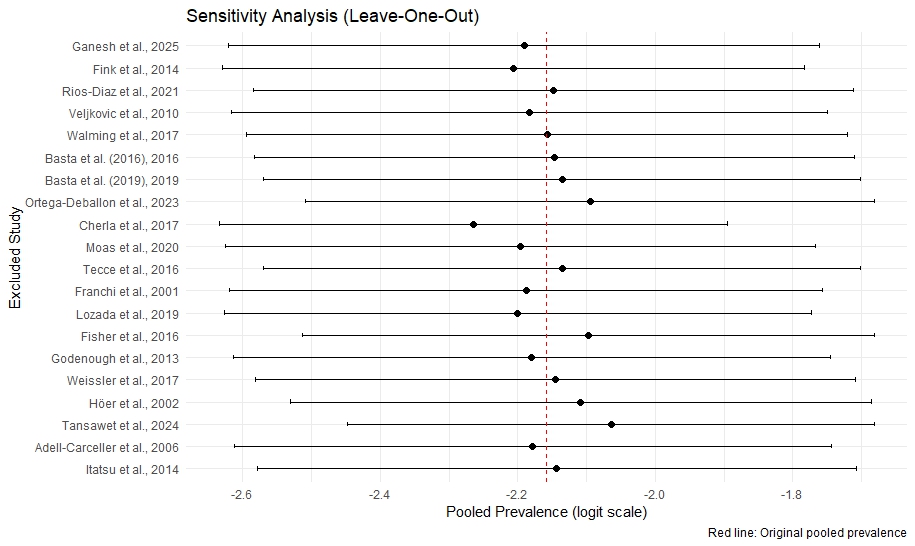


**Figure B.** Funnel plot of IH rates


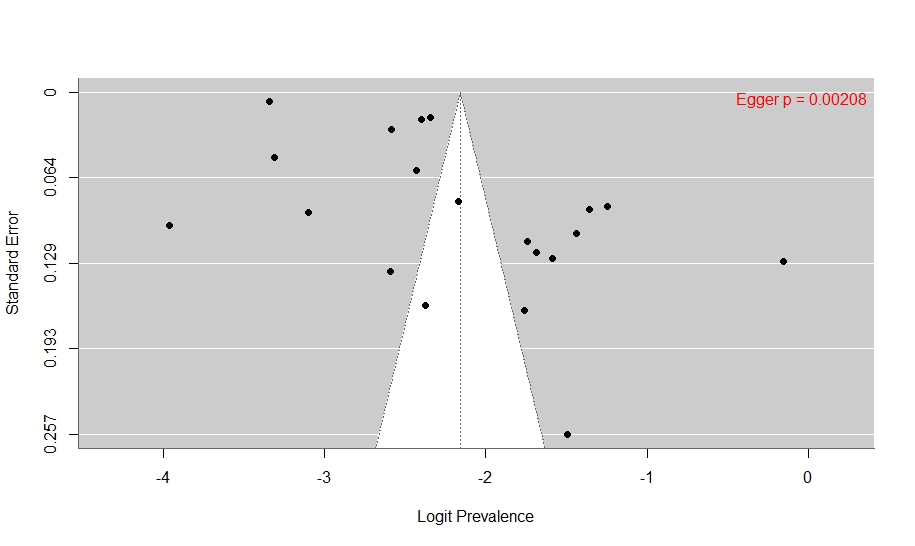


**Appendix 3. Risk of Bias**

| **Table A. Summary of Risk of Bias Assessment Using ROBINS-I Tool** | | | | | | | | | |
| --- | --- | --- | --- | --- | --- | --- | --- | --- | --- |
| **Study Title** | **Confounding** | **Selection** | **Classification** | **Deviations** | **Missing Data** | **Measurement** | **Reporting** | **Overall Risk** | **Quality** |
| Ganesh et al., 2025 | 3 | 2 | 1 | 1 | 2 | 1 | 1 | 3 (Serious) | Low |
| Fink et al., 2014 | 2 | 1 | 1 | 2 | 3 | 1 | 1 | 3 (Serious) | Moderate |
| Rios-Diaz et al., 2021 | 3 | 2 | 1 | 1 | 2 | 1 | 1 | 3 (Serious) | Low |
| Veljkovic et al., 2010 | 2 | 1 | 1 | 1 | 2 | 1 | 1 | 2 (Moderate) | Moderate |
| Walming et al., 2017 | 3 | 2 | 1 | 1 | 2 | 1 | 1 | 3 (Serious) | Low |
| Basta et al., 2016 | 2 | 1 | 1 | 2 | 3 | 1 | 1 | 3 (Serious) | Moderate |
| Basta et al., 2019 | 2 | 1 | 1 | 1 | 2 | 1 | 1 | 2 (Moderate) | Moderate |
| Ortega-Deballon et al., 2023 | 3 | 2 | 1 | 1 | 1 | 1 | 1 | 3 (Serious) | Moderate |
| Cherla et al., 2017 | 2 | 1 | 1 | 1 | 2 | 1 | 1 | 2 (Moderate) | Moderate |
| Moas et al., 2020 | 2 | 1 | 1 | 1 | 1 | 1 | 1 | 1 (Low) | High |
| Tecce et al., 2016 | 3 | 2 | 2 | 2 | 2 | 1 | 1 | 3 (Serious) | Moderate |
| Franchi et al., 2001 | 3 | 2 | 1 | 1 | 2 | 1 | 1 | 3 (Serious) | Moderate |
| Lozada et al., 2019 | 2 | 1 | 1 | 2 | 1 | 1 | 1 | 2 (Moderate) | Moderate |
| Fisher et al., 2016 | 3 | 1 | 1 | 2 | 2 | 1 | 1 | 3 (Serious) | Moderate |
| Goodenough et al., 2013 | 2 | 1 | 1 | 2 | 2 | 1 | 1 | 3 (Serious) | Moderate |
| Weissler et al., 2017 | 2 | 1 | 1 | 1 | 2 | 1 | 1 | 2 (Moderate) | Moderate |
| Höer et al., 2002 | 3 | 2 | 1 | 2 | 1 | 2 | 1 | 3 (Serious) | Moderate |
| Tansawet et al., 2024 | 2 | 1 | 1 | 1 | 2 | 1 | 1 | 2 (Moderate) | Moderate |
| Adell-Carceller et al., 2006 | 3 | 2 | 1 | 1 | 2 | 1 | 1 | 3 (Serious) | Moderate |
| Itatsu et al., 2014 | 2 | 1 | 1 | 1 | 2 | 1 | 1 | 2 (Moderate) | Moderate |
| *ROBINS-I: Risk Of Bias In Non-randomized Studies – of Interventions. This tool assesses bias across seven domains in non-randomized studies.* | | | | | | | | |  |
| *The overall risk of bias corresponds to the highest risk level observed in any domain.* | | | | | | | | | |
| **Low risk** → Comparable to a well-performed randomized trial. | | | |  |  |  |  |  |  |
| **Serious risk** → Important problems in at least one domain. | | | | **Critical risk** → Study is too problematic to provide any useful evidence. | | | | | |

**Moderate risk** → Sound for a non-randomized study, but not comparable to a randomized trial.

**Figure A. ROBINS-I Traffic-light plot**


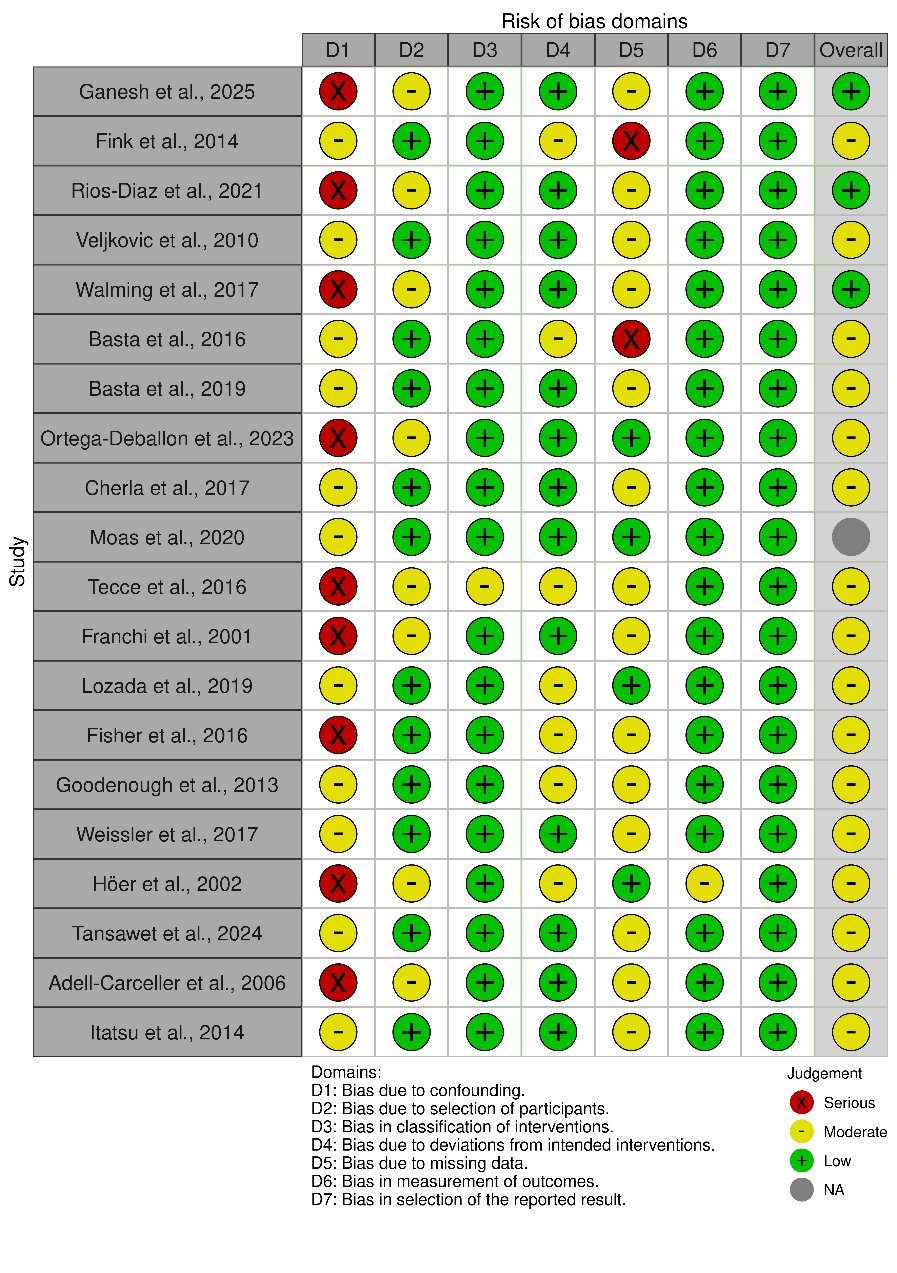


**Figure B. ROBINS-I Summary Plot**


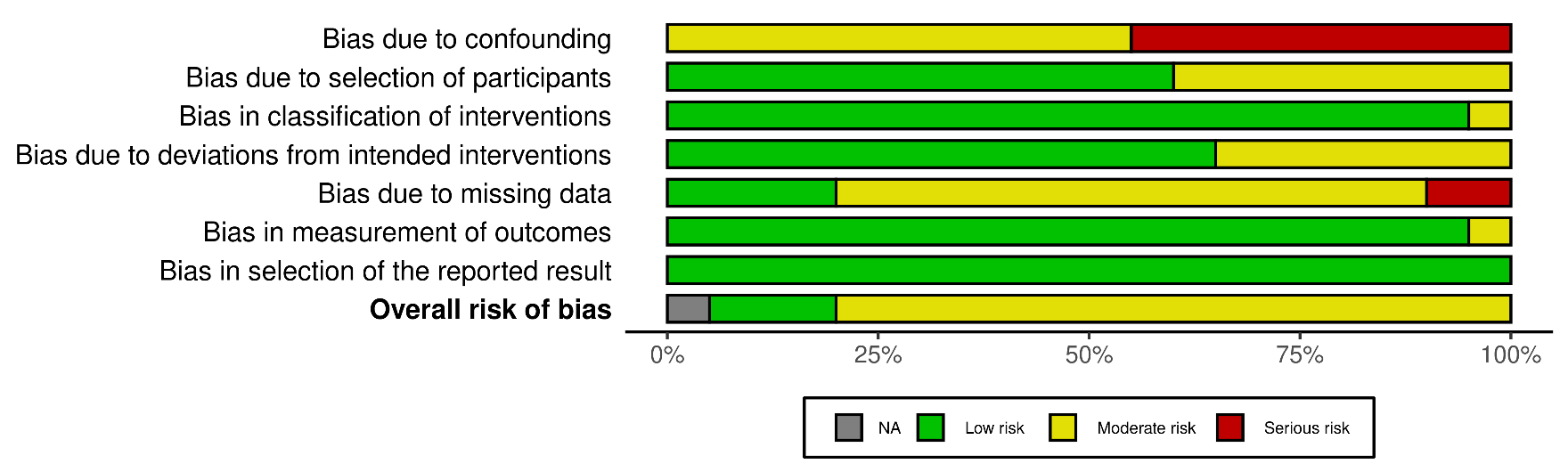

Supplement: Supplementary file 1 [file Supplementaryfile1.docx]
